# Isolation of a nanobody specific to the PstS-1 protein and evaluation of its immunoreactivity with structural components of *Mycobacterium tuberculosis* granuloma

**DOI:** 10.3389/fimmu.2025.1684904

**Published:** 2025-12-16

**Authors:** Yogesh P. Dhekale, Kumarasamy Jothivel, Samar Kumar Ghorui, Gagan Deep Gupta, Shweta Singh, Nawab Singh Baghel, Savita Kulkarni, Pramod Kumar Gupta

**Affiliations:** 1 Molecular Immunology and Tuberculosis Section, Radiation Medicine Centre, Bhabha Atomic Research Centre, TMH Annexe Building, Mumbai, India; 2 Homi Bhabha National Institute, Mumbai, India; 3 National Research Centre on Camel, Bikaner, India; 4 Protein Crystallography Section, Bhabha Atomic Research Centre (BARC), Mumbai, India; 5 Radiation Medicine Centre, Bhabha Atomic Research Centre, Tata Memorial Hospital (TMH) Annexe Building, Mumbai, India

**Keywords:** tuberculosis, TB granuloma, PstS-1, nanobody, T7 phage display, molecular imaging

## Abstract

*Mycobacterium tuberculosis* (Mtb) causes infectious granulomatous disease tuberculosis (TB), and existing *in vitro* TB-diagnosis is insensitive for extrapulmonary TB (EPTB) as well as paucibacillary TB due to low bacillary load; therefore, alternative non-invasive molecular imaging-based diagnostic tools are urgently required. Within TB granulomas, foci of Mtb secreted antigens anchored on the surface of either bacilli or host cells may serve as targetable biomarkers for antibody based molecular imaging of TB. Nanobody is better suited over conventional antibody or fragment derivatives for molecular imaging due to its quick localization in target tissue and rapid clearance from off-target organs. Here, we report the production of a high affinity nanobody against PstS-1 protein of Mtb which helps bacilli in phosphate uptake as well as host cell adhesion. C8 nanobody (C8Nb) was isolated from a phage displayed nanobody library which was constructed from a camel immunized with secreted proteins of Mtb. C8Nb was characterized *in vitro* and *in vivo* for immunoreactivity against PstS-1 protein. The ability of C8Nb to bind the PstS-1 protein, associated with the surface of Mtb bacilli or adhered on the macrophages, and its localization around BCG cells injected intramuscularly into mice, demonstrate its potential in the development of molecular imaging-based diagnostic tools for TB.

## Introduction

Tuberculosis (TB), caused by airborne pathogen *Mycobacterium tuberculosis* (Mtb), remains ancient and one of the deadliest diseases of mankind. In the year 2023, World Health Organization (WHO) estimated 10.8 million new cases and 1.25 million deaths related to TB. Out of 10.8 million estimated cases, only 8.2 million TB patients were diagnosed; leaving behind a steadily narrowing global gap of 2.6 million cases between the estimates and notifications (1). TB is a preventable as well as curable disease with proper medication; however, obstacles like variable efficacy of BCG vaccine (the only licensed vaccine), delay in decisive diagnosis, prolonged anti-TB drug therapy, and emergence of drug resistance are affecting the rate of reduction in TB incidences and deaths, which obscures the goals of ‘End TB Strategy’ in high TB burden countries (2). In the TB control program, diagnosis is a key component, as the early and precise diagnosis can promote prompt initiation of treatment to reduce the further disease transmission, damage to the infected tissue and development of drug resistance (3). Algorithm of TB diagnosis prescribes various tandem tests depending on the outcome of tests and alignment of the symptoms with TB. The cost-effective and rapid sputum-microscopy has been serving TB diagnosis for more than a century. However, when the bacillary load falls below 10^4^ bacilli/ml of sputum, the sensitivity of microscopy is significantly reduced (4). Chest X-ray examination is a very well-established primary screening tool which provides anatomical information necessary for the management of TB suspects (5). However, due to inconsistency in the extent of TB related tissue level abnormalities, which is governed by the competency of immune system (6), and their overlap with other clinical conditions (7), further examination is required to confirm the radiological findings suspecting TB. In the last decade, nucleic acid amplification-based diagnostic tests (NAATs) have revolutionized the detection of Mtb and drug resistance with their greater specificity and sensitivity along with shorter turnaround time. However, smear-negative paucibacillary TB in immunocompromised individuals carrying HIV co-infection as well as pediatric population is inadequately diagnosed by these tests (8, 9), which is responsible for ~16% of TB transmissions (10). TB culture test, which has turnaround time of few weeks, remains gold standard especially when rapid tests fail to detect Mtb. Despite plethora of available diagnostic tests, in year 2023, out of 8.2 million diagnosed TB patients, only 63% cases were bacteriologically confirmed, while 37% of TB patients were diagnosed clinically and initiated with empirical treatment to bring down the gap between TB case estimates and notifications to 2.6 million (1).

Immune cells such as macrophages, monocytes, neutrophils, dendritic cells, NK cells and lymphocytes are recruited to the site of infection, where these cells surround free as well as phagocytosed Mtb bacilli, and produce TB hallmark lesion called granuloma (11). Detection of TB granulomas has been explored using anatomical imaging through chest X-ray, ultrasound, MRI and CT, however, these techniques are not specific to TB lesions (12, 13). Active metabolism in Mtb bacilli as well as immune cells of granuloma has been explored as TB-diagnostic biomarkers through minimally invasive PET/CT and SPECT imaging. Various radiolabeled tracers, which are based on the precursor metabolites or antibiotic drugs, have shown potential in TB granuloma detection, however, their nonspecific nature limit their use only up to the disease staging, treatment response monitoring and predicting relapse (14). Structurally, TB granulomas are made up of aggregates of organized immune cells surrounding Mtb bacilli at the core (11). Growing Mtb actively secretes various proteins which promote extracellular as well as intracellular growth of pathogen (15). These proteins accumulate in the granulomas through their interactions with pathogen cell wall or host cell surface receptors (16). Presuming sufficient concentration of accumulated antigens within the granulomatous infection site, antibodies raised against such secreted antigens have been explored for molecular imaging of TB granulomas. In the earlier attempts of immunoscintigraphy, TB lesions were imaged using radiolabeled intact antibodies (17, 18) as well as F(ab’)_2_ fragments (19) specific to mycobacterial antigens. However, imaging contrast was produced only after days to weeks post tracer administration. Molecular size of tracer determines its kinetics of accumulation in the target tissue and clearance from the background, which ultimately decides optimum timing for imaging (20). Currently, various small sized recombinant proteins with faster pharmacokinetics are being developed for tumor imaging and therapy. Nanobody is a ~15kDa recombinant protein derived from variable domain of heavy chain antibody produced by camelids (21). In contrast to intact IgG antibodies, which remain in the circulation for longer period of time due to their slow extravasation and binding with Fc gamma receptors, compact nanobodies offer better tissue penetration and faster clearance from the circulation to produce imaging contrast within few hours of the administration (20, 22). This feature of nanobody will help diagnosing EPTB including TB meningitis, as the nanobodies have shown the ability to cross the blood brain barrier (23). Further, use of recombinant antibodies lacking Fc domain mitigates their nonspecific accumulations around the pathogen or host cells due to the interactions between Fc domain and Fc gamma receptors (24–26). Use of pathogen specific antibodies may help in discriminating infection from sterile inflammations and malignancies.

Cell wall associated PstS-1 protein of Mtb is a phosphate binding subunit of Phosphate Specific Transport (PST) system (27–30). PstS-1 protein remains anchored in the Mtb cell wall, through its hydrophobic tail and participates in the phosphate transport (31, 32). Additionally, PstS-1 protein is also known to play role in Mtb infection through its interactions with various Pattern Recognition Receptors (PRRs) including mannose receptor (MR), TLR2 and TLR4 (30, 33, 34). Therefore, PstS-1 protein capable of accumulating in the TB granulomas through its interactions with Mtb cell wall or PRRs expressed on the immune cells forming granuloma appears to be a suitable target for nanobody based molecular imaging of TB granulomas.

In the present study, we have constructed a phage displayed nanobody library (henceforth mentioned as nanobody library) and used for isolation of nanobodies specific to PstS-1 protein of Mtb. Binding specificity, affinity and ability of lead C8Nb to bind PstS-1 protein present on the surface of either Mtb bacilli or macrophages were evaluated using *in vitro* binding assays. Further confirmation of *in vivo* localization of ^125^I labelled C8Nb (^125^I-C8Nb) around BCG cells injected through intramuscular route into mouse model suggests that the C8Nb may be useful in the development of molecular imaging-based modalities for precise diagnosis, staging as well as evaluation of treatment response in TB.

## Materials and methods

### Materials and reagents

Freund’s adjuvants, complete (Cat. No. F 5881) and incomplete (Cat. No. F 5506), were purchased from Sigma-Aldrich. EcoRI, HindIII and T4 DNA Ligase were purchased from New England Biolabs. For nanobody display library construction, T7Select 1–1b cloning Kit (Cat. No. 70010) was purchased from Merck Millipore. Mouse anti-T7 tail fiber mAb (anti-T7TF mAb, Cat. No. 71530) was procured from Merck Millipore. Rabbit anti-camelid V_H_H antibody-HRP conjugate (Cat. No. A01861) and rabbit anti-camelid V_H_H cocktail-iFluor555 (Cat. No. A02020) were purchased from GenScript. Oligonucleotides used in this study were ordered from Eurofins genomics (**Supplementary Table S1**). Iodination grade Na^125^I was supplied by BRIT, Mumbai, India. Sensor Chip CM5 (Cat. No BR100012) was procured from Cytiva.

### Antigen preparation

Entire secreted proteins of laboratory strain Mtb H37Rv were prepared by concentrating the culture filtrate as described previously (35). Briefly, Mtb was inoculated in Middlebrook 7H9 broth enriched with ADC growth supplement and 0.05% Tween 80 and incubated at 37 °C in shaker incubator. Log phase culture of Mtb was centrifuged at 7800×g for 15 min and pellet was washed with phosphate buffered saline (1xPBS). Further, Mtb pellet was resuspended in 1xPBS (20ml) and suspension was transferred into 50ml falcon tube containing 15–20 sterile glass beads (3mm diameter). Tube was vortexed at maximum speed for 5 min and kept standing for 5 min at room temperature (RT). The upper half of the bacterial suspension was passed through 26-gauge needle for 8–10 times and optical density at 600nm (OD_600_) was adjusted to ~1. Subsequently, Mtb was inoculated at the density of 10^6^ bacilli/ml in synthetic Sauton’s medium enriched with 0.5% glucose, 0.5% sodium pyruvate, and cultured at 37 °C in orbital shaker incubator (36). After 14 days of growth, culture was centrifuged at 12,800×g for 30 min at 4 °C. The clear supernatant was collected and saturated with ammonium sulphate (80%). Following overnight incubation at 4°C, protein precipitate was spun down at 20,000×g for 20 min and resuspended in 50ml 1xPBS. Further, insoluble debris were removed by centrifugation and the clear supernatant was filter sterilized through 0.22µm membrane. Total protein was concentrated and washed with 1xPBS on 10kDa cut-off membrane concentrator (37–39). To ascertain sterility, 100µl protein sample was streaked on Middlebrook 7H10 media and plate was incubated at 37°C for 3 weeks. To produce recombinant PstS-1 (rPstS-1) protein, *E. coli* BL21(DE3) expressing PstS-1 protein of Mtb was cultured in Terrific-broth at 37 °C, and at OD_600_ ~1, the protein expression was induced with final concentration of 0.5mM IPTG at 20°C. After 16 h induction, bacterial biomass was probe sonicated in lysis buffer (5mM imidazole, 50mM phosphate and 300mM NaCl, pH 8) supplemented with PMSF (1mM) and lysozyme (0.2mg/ml). Lysate was centrifuged at 20000×g for 45 min and clarified supernatant was incubated with Ni-NTA agarose beads for 1 h at RT on tube roller. Protein bound Ni-NTA agarose beads were washed with 100ml lysis buffer and protein was eluted with 150mM imidazole. Protein purity was assessed on 14% SDS-PAGE.

### Camel immunization

A two and half year old healthy dromedary of Kutchi breed (K-136, ♂) maintained at National Research Centre on Camel (NRCC), Bikaner, India was immunized with Mtb antigens. About 10ml of pre-immunization bleed was collected for evaluating the presence of preexisting immune profile against antigens of interest. Further, 0.5ml of Mtb antigens (500μg/dose, in 1xPBS) was emulsified in equal volume of Freund’s complete adjuvant. One side of the camel shoulder area around prescapular region was shaved, cleaned, and disinfected to administer the emulsion. Using a 23-gauge needle, small aliquots of the emulsion were administered subcutaneously into multiple sites. Subsequent boosters were prepared as stated earlier in Freund’s incomplete adjuvant and administered at 21-day intervals. On the seventh day post fourth booster, about 50ml of blood was drawn from jugular vein and peripheral blood mononuclear cells (PBMCs) were isolated using Histopaque-1077, dissolved in 5ml TRIzol reagent, and stored at -80 °C until further use. Presence of heavy chain antibodies specific to PstS-1 protein in the camel serum was assessed using ELISA. Briefly, 100µl of rPstS-1 protein (2μg/ml in 0.1M bicarbonate buffer, pH 9.6) was dispensed into microtiter wells at 37 °C for 1 h. Wells were washed thrice with 1xPBS containing 0.05% Tween 20 (1xPBST) and blocked with 200μl of 2% BSA for 30 min. Thereafter, 100µl of each camel serum dilution from nine consecutive two-fold dilutions were incubated in wells for 1 h at 37 °C. Wells were washed thrice with 1xPBST and 100µl of rabbit anti-camelid V_H_H antibody-HRP conjugate (1:10,000) was incubated in wells for 1 h at 37 °C. Subsequently, wells were washed four times with 1xPBST and 100µl ready to use TMB substrate was added to the wells and plate was incubated at RT in dark. Color development was stopped with 100µl of 1N HCI and absorbance was measured at 450nm. The procedures involving camel handling were approved by Institutional Animal Ethics Committee (IAEC), ICAR-NRCC, India (Sanction Number: NRCC/PME/6(141)/2000-Tech/Vol-IIPBMC) and were performed by professional veterinarians as per the guidelines.

### Nanobody library construction

T7 phage displayed immune nanobody library against secretory proteins of Mtb was constructed by following the previously reported methodology (21). Briefly, total RNA was extracted from PBMCs and subjected to cDNA synthesis by reverse transcription using Hyperscript First strand synthesis kit (APExBIO). Camel produces both conventional (IgG1) as well as heavy chain antibodies (IgG2 and IgG3), therefore two-step PCR approach was adopted to amplify and separate V_H_H gene fragments from VH amplicons. In the first-step, IgG-PCR was performed on cDNA using previously published primers, i.e., CH2FORTA4 and VHBACKA6 (**Supplementary Table S1**), and amplicons of all IgG subclasses were separated by agarose gel electrophoresis. Due to presence of CH1 domain, the IgG1 subclass produces 900bp size amplicons, while IgG2 and IgG3 subclasses lacking CH1 domain produce amplicons of 690bp and 620bp, respectively, where the length difference arises mainly due to variation in the length of hinge coding regions (**Supplementary Figure S1**). In the second-step, gel purified 690bp and 620bp amplicons were used as DNA template in V_H_H PCR to amplify the nanobody coding regions between FR1-FR4 (Primers- FORECORI and BACKHINDIII, **Supplementary Table S1**). V_H_H amplicons were double digested with EcoRI and HindIII restriction endonucleases. Subsequently, 11ng of V_H_H amplicons and 500ng of T7 phage vector DNA arms (3:1 molar ratio) with cohesive ends were incubated with T4 DNA Ligase at 16 °C. Next day, a PCR with primers specific to vector arms (Primers: T7SelectUp and T7SelectDown, **Supplementary Table S1**) was performed to confirm the ligation. For *in vitro* phage packaging, 5μl of ligation reaction mixture was mixed with 25μl of T7 phage packaging extract and incubated for 2 h at 22 °C. Phage packaging reaction was terminated by adding 270μl of LB medium and number of assembled infective phages was determined by plaque assay. Prior to bio-panning, whole library was amplified in liquid culture to express and display the nanobodies on T7 phage capsid and stored at -80 °C.

### Enrichment and selection of nanobodies specific to PstS-1 protein

High affinity nanobodies specific to PstS-1 protein were enriched and screened by the bio-panning strategy reported earlier from our laboratory (21). For bio-panning, 100µl of rPstS-1 protein (2μg/ml in 0.1M bicarbonate buffer, pH 9.6) was incubated overnight in microtiter wells at 4 °C, washed thrice with 1xPBS and blocked with 200μl of 2% BSA solution for 30 min at 37 °C. Phage library was mixed with equal volume of 4% BSA for 15 min at RT and 100μl of BSA blocked library was added to wells for 2 h at 37 °C. Further, wells were washed 10 times with 1xPBST and bound phages were either eluted in 100μl of 1% SDS for enumeration by plaque assay or amplified *in situ* by adding fresh culture of host *E. coli* Rosetta-gami B5615. Amplified phages were transferred to 40ml culture of *E. coli* Rosetta-gami B5615 and phage amplification was continued at 37 °C. In each cycle, BSA coated wells were processed similarly as negative controls. To increase washing stringency for enrichment of strong binders, 0.5M and 1M NaCl wash were given following ten 1xPBST washes in 2^nd^ and 3^rd^ cycles of bio-panning, respectively. After 3 rounds of enrichment, monoclonal phage lysates were prepared for randomly selected 30 nanobody clones. Immunoreactivity of monoclonal phage lysates was assessed using a two-step screening approach. In the first assay, monoclonal phage lysates were incubated with the coated antigen, while in the second assay, phages were captured via their tail using coated anti-T7TF mAb, and reacted with ^125^I-labelled rPstS-1 protein. For the first assay, 300μL of rPstS-1 protein was coated overnight into polystyrene star tubes at 4 °C. Unbound protein was discarded and tubes were blocked with 2% BSA solution. Further, monoclonal phage lysates were blocked with equal volume of 4% BSA and 300μl of each clone was added to rPstS-1 protein coated tubes in triplicate for 2 h at 37 °C. Tubes were washed thrice with 1xPBST and incubated with 300μl of ^125^I-anti-T7TF mAb (~1×10^5^ Counts Per Minute (CPM)) at RT. Following 2 h incubation, tubes were washed thrice with 1xPBST and bound tracer was measured in gamma-photon counter (PC-RIA. MAS STRATEC). Ten nanobody clones, selected based on consistent binding, were subjected to next assay, where 100μl of each phage lysate was incubated in detachable wells coated with anti-T7TF mAb for 2 h at 37 °C. Wells were washed thrice with 1xPBST and then reacted with 100μl of ^125^I-labelled rPstS-1 protein (~1×10^5^ CPM) at RT. Wells were washed thrice with 1xPBST after 2 h of incubation, and bound activity was measured in gamma-photon counter. Subsequently, nanobody genes amplified using PCR (Primers: T7SelectUp and T7SelectDown) were subjected to Sanger sequencing. After nucleotide sequence analysis, nanobody clone 8 (C8Nb) was selected for further studies. The gene sequence of the C8Nb was submitted to DDBJ (Accession no. LC899821) (**Supplementary Material**).

### Cloning, overexpression and purification of C8Nb

C8Nb was overexpressed in *E. coli* BL21(DE3) and purified using multiple protein purification methods. Codon-optimized 399bp gene encoding C8Nb with C-terminal 6×His tag was synthesized and cloned into pET-22b (+) expression vector. The plasmid construct was electroporated into *E. coli* BL21(DE3) and transformants were selected on ampicillin (100µg/mL) added media. Single colony was inoculated in 10ml Terrific-broth supplemented with ampicillin (100µg/mL) and cultured overnight at 37 °C. Overnight grown inoculum (5ml) was added to 1 liter of Terrific-broth, incubated at 37 °C, and once OD_600_ reached to ~1, the protein expression was induced with final concentration of 1mM IPTG at 20 °C for 16 h. Culture was centrifuged at 2,000×g for 15 min at 4 °C and bacterial pellet was resuspended in 40ml lysis buffer (5mM imidazole, 50mM phosphate and 300mM NaCl, pH 8) supplemented with PMSF (1mM) and lysozyme (0.2mg/ml). Bacterial suspension was probe sonicated (5 sec on/off) on ice for 30 min. Lysate was centrifuged at 20,000×g for 45 min at 4 °C and clear supernatant was loaded on Ni-NTA agarose column equilibrated with lysis buffer. After washing the column with lysis buffer, bound protein was eluted in 300mM imidazole. Selected protein fractions containing C8Nb were concentrated on 10kDa cut-off concentrator and resuspended in buffer (10mM NaCl, 20mM Tris pH, 7.8) compatible with anion exchange chromatography. Protein sample was loaded on equilibrated DEAE cellulose column and unbound C8Nb (Isoelectric point (pI) 7.8) from flow through was concentrated. In the next step, protein sample was gel filtered through Superdex 75 Increase 10/300 GL column equilibrated with 1xPBS to remove remaining impurities, and C8Nb fractions were stored at 4 °C. After each purification step nanobody purity was evaluated on 14% SDS-PAGE.

### Determination of equilibrium dissociation constant (*K_D_*) using saturation binding ELISA

Affinity of C8Nb was measured by ELISA using previously described protocol with minor modifications (40). Briefly, 100μl of rPstS-1 protein (2μg/ml, pH) was dispensed per well into microtiter plate and incubated for 1 h at 37 °C. Following three 1xPBS washes, wells were incubated with 2% BSA for 30 min at 37 °C, washed twice with 1xPBST and incubated with 100µl of each dilution of seven consecutive tenfold dilutions of C8Nb (2000nM-0.002nM) for 1 h at 37 °C. After four 1xPBST washes, wells were incubated with 100µl of rabbit anti-camelid V_H_H antibody-HRP conjugate (1:10,000) for 1 h at 37 °C, washed again for five times with 1xPBST, and developed with TMB substrate. The assay was performed in triplicate (*n=*3). Signal intensities were plotted as function of C8Nb concentrations in GraphPad Prism and data were analyzed using nonlinear regression function (One site–Specific binding with Hill slope) (41).

### Binding kinetics of C8Nb by surface plasmon resonance

Kinetics of binding between C8Nb and rPstS-1 protein were analyzed on Biacore X100 SPR system (GE Healthcare) at 25 °C (42). Briefly, rPstS-1 protein was covalently immobilized on CM5 sensor chip using a standard amine coupling kit (GE Healthcare). C8Nb, diluted in running buffer (10mM HEPES pH 7.0, 100mM NaCl, 0.005% P20), was injected onto the antigen-bound sensor chip at the increasing concentrations (ranging from 5nM to 240nM) in multi-cycle kinetic assays. Sensor chip was regenerated after every cycle with glycine-HCl buffer (10mM, pH 2.5) and the binding kinetics were repeated thrice on the same chip. The acquired data were processed using Biacore evaluation software V2.0.2 (GE Healthcare), and the kinetic rate constants *k_a_*, *k_d_* and *K_D_* were determined using a 1:1 binding model utilizing the SPR data obtained for nanobody concentration such as 5nM, 10nM, 20nM, 40nM and 60nM, where the sensorgrams have sufficient curvature.

### Binding specificity evaluation by immunoblotting

Mycobacterial strains (Mtb H37Rv, BCG (Moscow) and *M. smegmatis)* were cultured in Middlebrook 7H9 broth added with ADC growth supplement and 0.05% Tween 80 at 37 °C. *E. coli* was cultured in Terrific-broth at 37 °C in shaker incubator. Bacterial cells were harvested from log phase cultures and two 1xPBS wash were given. Cells resuspended in 1ml 1xPBS were then transferred to lysing matrix B (MP Biomedicals). Bacterial lysis was performed using preset protocols in FastPrep-24™ 5G bead beating grinder (MP Biomedicals). Lysate was centrifuged at 20,000×g for 45 min, supernatant was collected and protein concentration was determined by Bradford assay. Protein samples (2μg rPstS-1 protein or 100μg of crude protein mixture from Mtb, BCG, *M. smegmatis* and *E. coli*) were electrophoresed through 14% SDS-PAGE and then electroblotted on PVDF membrane. Blotted membrane was blocked overnight in 5% skimmed milk in 1xPBS, incubated with C8Nb (2μg/ml in 2% BSA) at RT for 3 h, washed thrice (5ml, 1xPBST for 15 min) and incubated with rabbit anti-camelid V_H_H antibody-HRP conjugate (1:10,000) for 2 h at RT. Further, membrane was washed with 1xPBST and developed with chemiluminescent substrate (CLS).

### ^125^I labelling of C8Nb

Radioiodination of C8Nb was done using Iodogen method as described previously (43). To prepare Iodogen coated tubes, 100µl of Iodogen (1mg/ml in CHCl_3_) was added to 1.5ml Eppendorf tubes and air dried to form Iodogen layer on tube wall. Fifty microliters of C8Nb (100µg/ml in 500mM phosphate buffer, pH 7.4) was dispensed to the bottom of Iodogen coated tube. Further, 4µl of ^125^I (~771µCi) activity was added to the same tube, mixed well, and incubated for 5 min at RT. Iodination was terminated by adding 200µl of 50mM phosphate buffer and reaction mixture was loaded on Sephadex G-25 (coarse) column. Subsequently, 2% BSA solution in 1xPBS was passed through the column, and elution fractions of 0.5mL were collected.

### ^125^I-C8Nb radioimmunoassay

100μl of rPstS-1 protein (2μg/ml, pH 9.6) was coated overnight in detachable microtiter wells at 4 °C. After 1xPBS wash, wells were blocked with 200μl of 2% BSA for 30 min at 37 °C and incubated with 100μl of ^125^I-C8Nb tracer (~1.19×10^5^ CPM). Tracer was used either in its normal form or spiked with unlabeled C8Nb (15μg/ml, spiked tracer). BSA coated wells were treated similarly as negative controls. The assay was performed in triplicate (*n=*3). After 1 h incubation at RT, wells were washed four times with 1xPBST and bound tracer was measured in gamma-photon counter.

### Immunoreactivity of ^125^I-C8Nb against PstS-1 protein present in the cell wall of drug sensitive as well as multi-drug resistant mycobacteria

Mycobacteria such as Mtb H37Rv, Mtb LAM, Mtb Beijing, BCG and *M. smegmatis* were cultured and processed to prepare single cell suspensions. *E. coli* was inoculated in terrific-broth and cultured at 37 °C. Single cell suspensions were washed twice with 1xPBS and OD_600_ was adjusted to ~1. One milliliter suspension of each strain was centrifuged at 7,800×g for 10 min and pellets were resuspended in 0.5ml 2% BSA at 37 °C. After 30 min blocking, cells were spun down and resuspended in 100μl of ^125^I-C8Nb (~1.2×10^5^ CPM) prepared in 2% BSA. Similar bacterial pellets were also treated with spiked tracer. After 30 min binding at RT, cells were pelleted down and washed twice with 1xPBS. Further, 0.5ml 10% formaldehyde was added on the top of pellets and bound tracer was measured in gamma-photon counter.

### Immunoreactivity of ^125^I-C8Nb with PstS-1 protein adhered on macrophages

Murine macrophage cell line RAW 264.7 was cultured under 5% CO_2_ in DMEM supplemented with 10% FBS and seeded in 12 well culture plate at density of 0.25×10^6^ cells/well. Next day, cells were washed with 1xPBS, fixed with 4% formaldehyde for 15 min at RT, followed by blocking with 2% BSA for 30 min at 37 °C. Subsequently, cells were incubated with protein samples (200µg of crude protein mixture prepared from Mtb, BCG, *M. smegmatis*, *E. coli* bacterial lysates or 2µg of rPstS-1 protein) resuspended in 200µl of blocking solution for 1 h at 37 °C. Cells were washed thrice with 1xPBS and 200µl of ^125^I-C8Nb (~2×10^5^ CPM) was added in each well. After incubating for 1 h at 37 °C, cells were washed thrice with 1xPBS and 200µl of 1% SDS was incubated in each well for 5 min at RT. The assay was performed in triplicate (*n=*3). Well contents were collected in tubes and bound tracer was measured in gamma-photon counter.

### Determination of PRR involved in macrophage binding with PstS-1 protein-C8Nb complex

Human monocyte cell line THP-1 (wild type,WT) was cultured under 5% CO_2_ in RPMI 1640 medium supplemented with 10% FBS at 37 °C. TLR-2 and TLR-4 knockout (KO) THP-1 cells (Invivogen) were cultured and maintained in RPMI 1640 medium under selection pressure of blasticidin (10µg/ml) and zeocin (100µg/ml). Approximately 0.5×10^6^ cells resuspended in 1ml complete medium containing Phorbol Myristate Acetate (PMA, 100ng/ml) were seeded in 12 well cell culture plate and incubated overnight at 37 °C. Next day, cells were washed twice with 1xPBS and fixed with 4% formaldehyde for 15 min at RT. In the next step, cells were washed thrice with 1xPBS and blocked with 0.5ml 2% BSA for 30 min at 37 °C. Further, blocking solution was discarded and cells were incubated with 2µg of rPstS-1 protein resuspended in 200µl of blocking solution for 1 h at 37 °C. Subsequently, wells were washed thrice with 1xPBS and 200µl of ^125^I-C8Nb (~2.3×10^5^ CPM) was added in each well. Similarly, to evaluate the role of MR in binding, WT THP-1 cells were treated with 200µg of Mtb protein mixture (with or without mannose, 1mg/ml), resuspended in 200µl 2% BSA. Spiked tracer was added to similarly treated control wells. After 1 h binding at 37 °C, cells were washed thrice with 1xPBS and 200µl of 1% SDS was added in each well for 5 min at RT. Further, well contents were collected and retained activity was measured in gamma-photon counter. Similarly, for fluorescence microscopy, cells were cultured and prepared in µ-slide 8 well glass bottom plate (Ibidi). Further, 100µl of rPstS-1 protein (10µg/ml) was added to each well and incubated at 37 °C for 1 h. Wells were washed thrice with 1xPBS and incubated with 100µl of C8Nb (1µg/ml) prepared in 2% BSA. After 1 h binding at 37 °C, cells were washed thrice with 1xPBS and incubated with 100µl of rabbit anti-camelid V_H_H cocktail-iFluor555 (1:500) for 1 h at 37 °C. Further, cells were washed thrice with 1xPBS and incubated with 100µl of DAPI stain (1µg/ml in 1xPBS) for 10 min at RT. Subsequently, after 1xPBS wash, 100µl of 2% formaldehyde was added to wells and images were acquired on Mica Microhub system (Leica) and analyzed using LAS X Software. (URL link: https://www.leica-microsystems.com/products/microscope-software/p/leica-las-x-ls/downloads/).

### *In vivo* localization of ^125^I-C8Nb around BCG cells

For *in vivo* localization study, 4–5 week old BALB/c mice were obtained from animal house, BARC, Trombay and maintained at animal house, RMC, Parel. Biodistribution study protocols were approved by IAEC, BARC, India (Sanction Number: BAEC/34/23, Dated 09/01/2024). Three days prior to the study, drinking water was replaced with potassium iodide (KI) solution (1g/L water). Single cell suspensions of *M. smegmatis* and BCG were prepared in 1xPBS, and ~5×10^8^ cell number was adjusted in 50µl of 1xPBS. Animals were anesthetized with ketamine (80mg/kg body weight, Intraperitoneal), and 50µl of BCG (right arm) and *M. smegmatis* (left arm) cell suspensions were injected through intramuscular route. Further, 75µl of ^125^I-C8Nb (~150ng C8Nb, 25.91µCi) was injected through catheterized tail vein. After 4 h, animals were euthanized with overdose of anesthesia (Ketamine, 80mg/kg; Xylazine, 5mg/kg body weight) followed by cervical dislocation, and limbs were collected for gamma-photon counting.

## Results

### Construction of nanobody library

**Figure 1:**
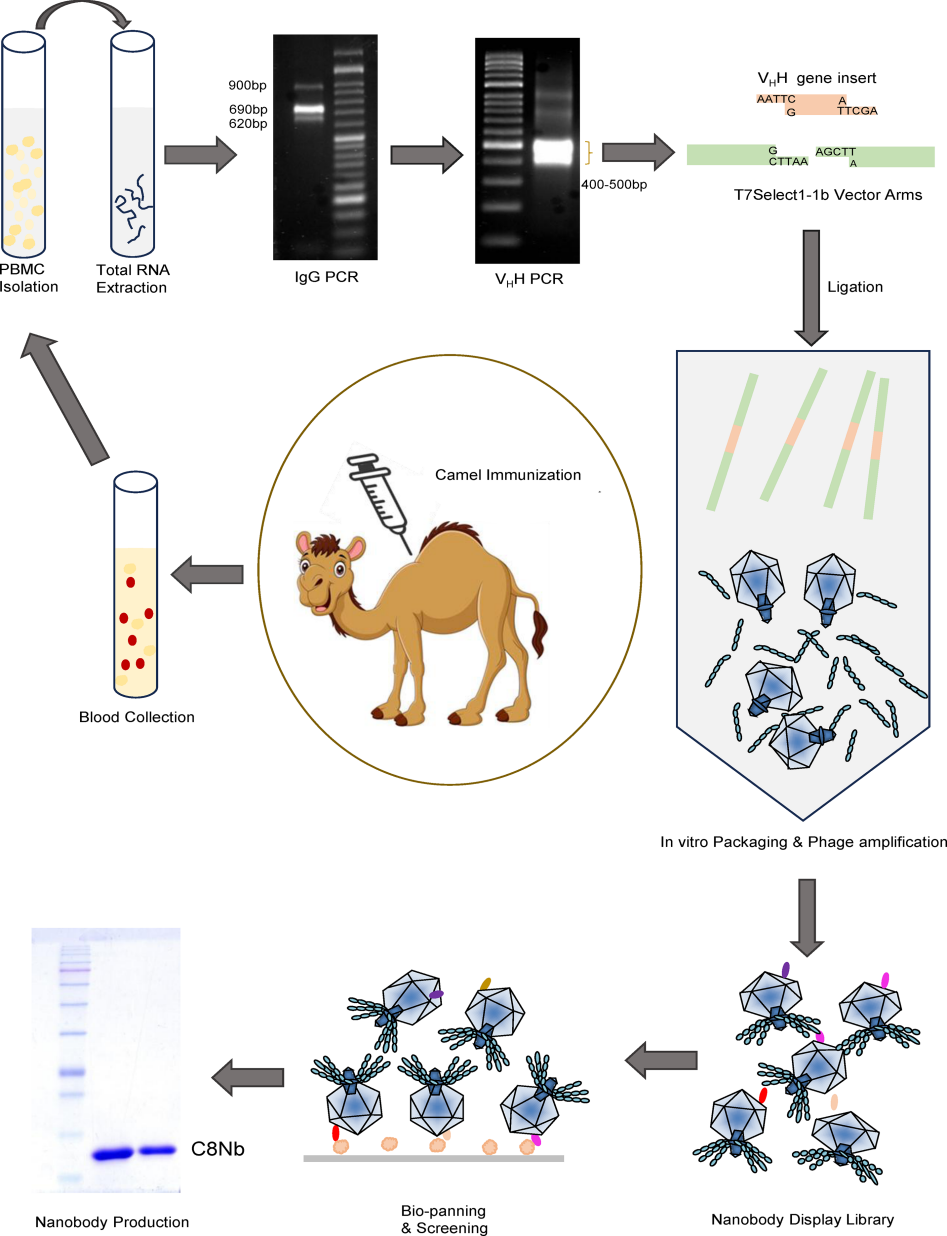
Schematic map depicting construction of nanobody display library and production of nanobody.

**Figure 2:**
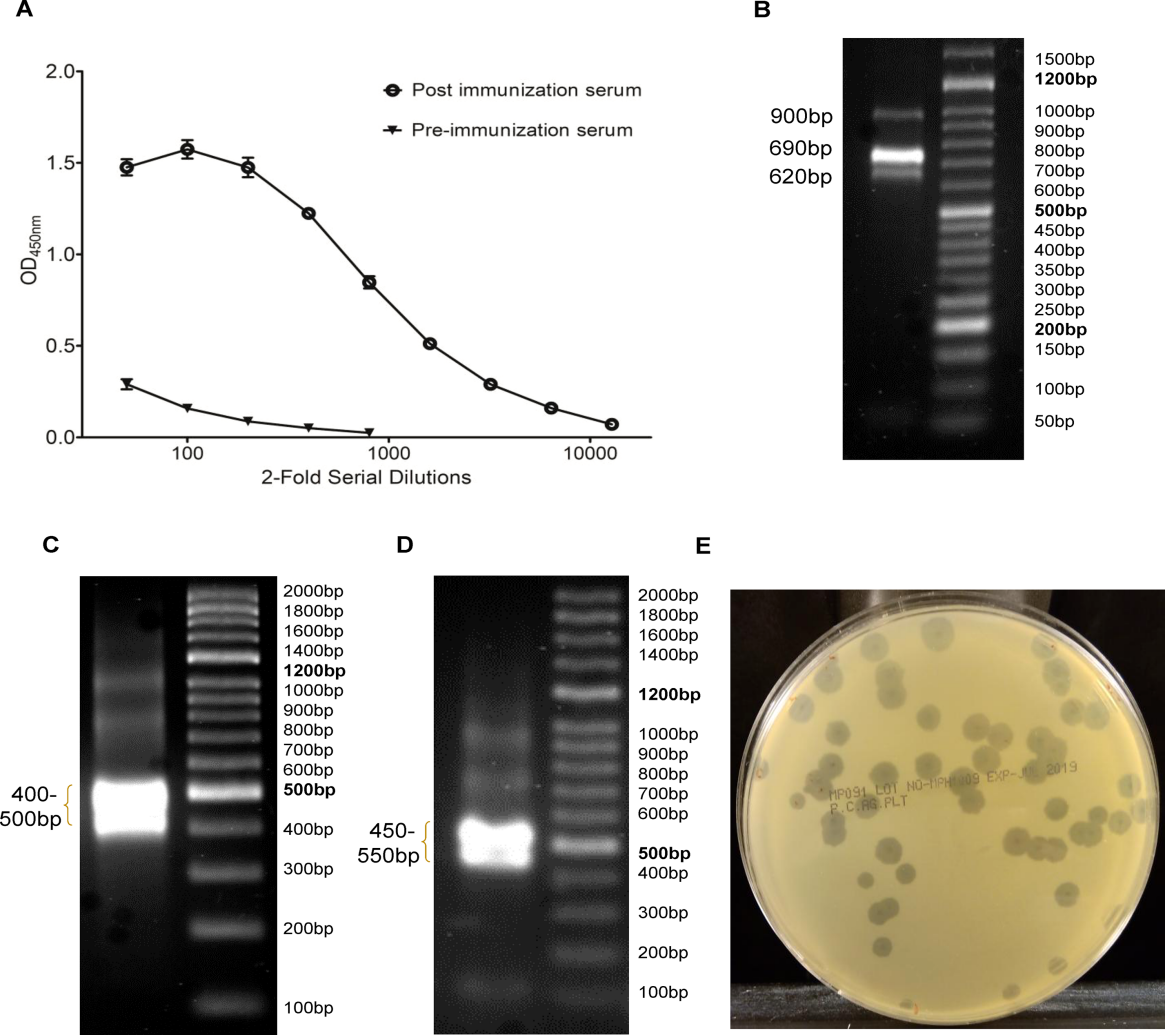
Nanobody display library construction. **(A)** Generation of heavy chain antibodies in camel serum was confirmed by ELISA, where ELISA wells coated with rPstS-1 protein were incubated with serially diluted camel sera and bound antibodies were measured by incubating rabbit anti-camelid V_H_H antibody-HRP conjugate. **(B)** In IgG PCR, genetic locus between FR1 region of variable domain and 5’ end of CH2 domain were amplified, which yielded 620bp, 690bp and 900bp size amplicons corresponding to IgG3, IgG2 and IgG1 subclasses, respectively. **(C)** In V_H_H PCR, primers amplified the region between FR1 and FR4, which produced a broad band of ~400bp-500bp size amplicons. **(D)** Vector based PCR (Primers: T7SelectUp and T7SelectDown) on ligation reaction mixture produced broad band of ~450bp-550bp size amplicons, which confirmed ligation between nanobody gene inserts and T7 vector DNA arms. **(E)** Nanobody library size (~2.5×10^5^ clones) was measured by PFU assay. Image shows clear plaques of T7 phages on lawn of *E*. *coli* Rosetta-gami B5615.

To construct nanobody display library, a healthy dromedary was immunized with mixture of Mtb secretory proteins. **Figure 1** depicts the schematic map for construction of nanobody display library and isolation of anti-PstS-1 C8Nb. Additional details on cloning involved in nanobody display using T7 phage system are given in **Supplementary Figure S1**. Following the last booster, camel seroconversion was confirmed by ELISA, where post immunization camel serum demonstrated generation of PstS-1 protein specific heavy chain antibodies (**Figure 2A**). cDNA was prepared using total RNA extracted from camel PBMCs and used for nanobody gene preparation in two step PCR. The IgG-PCR yielded a mixture of gene fragment amplicons (900bp, 690bp, and 620bp), which were separated by agarose gel electrophoresis, and the 690bp and 620bp amplicons were subsequently purified (**Figure 2B**). These purified amplicons served as DNA template in V_H_H PCR, which yielded a distinct broad band of approximately 400bp-500bp fragments (**Figure 2C**). Ligation reaction between EcoRI and HindIII digested V_H_H gene inserts and T7 phage vector DNA arms was validated through vector based PCR, which generated a broad band of 450bp-550bp size amplicons (**Figure 2D**). The subsequent *in vitro* phage packaging produced a library of ~2.5×10^5^ nanobody clones (**Figure 2E**).

### Isolation of PstS-1 protein specific nanobodies

**Figure 3:**
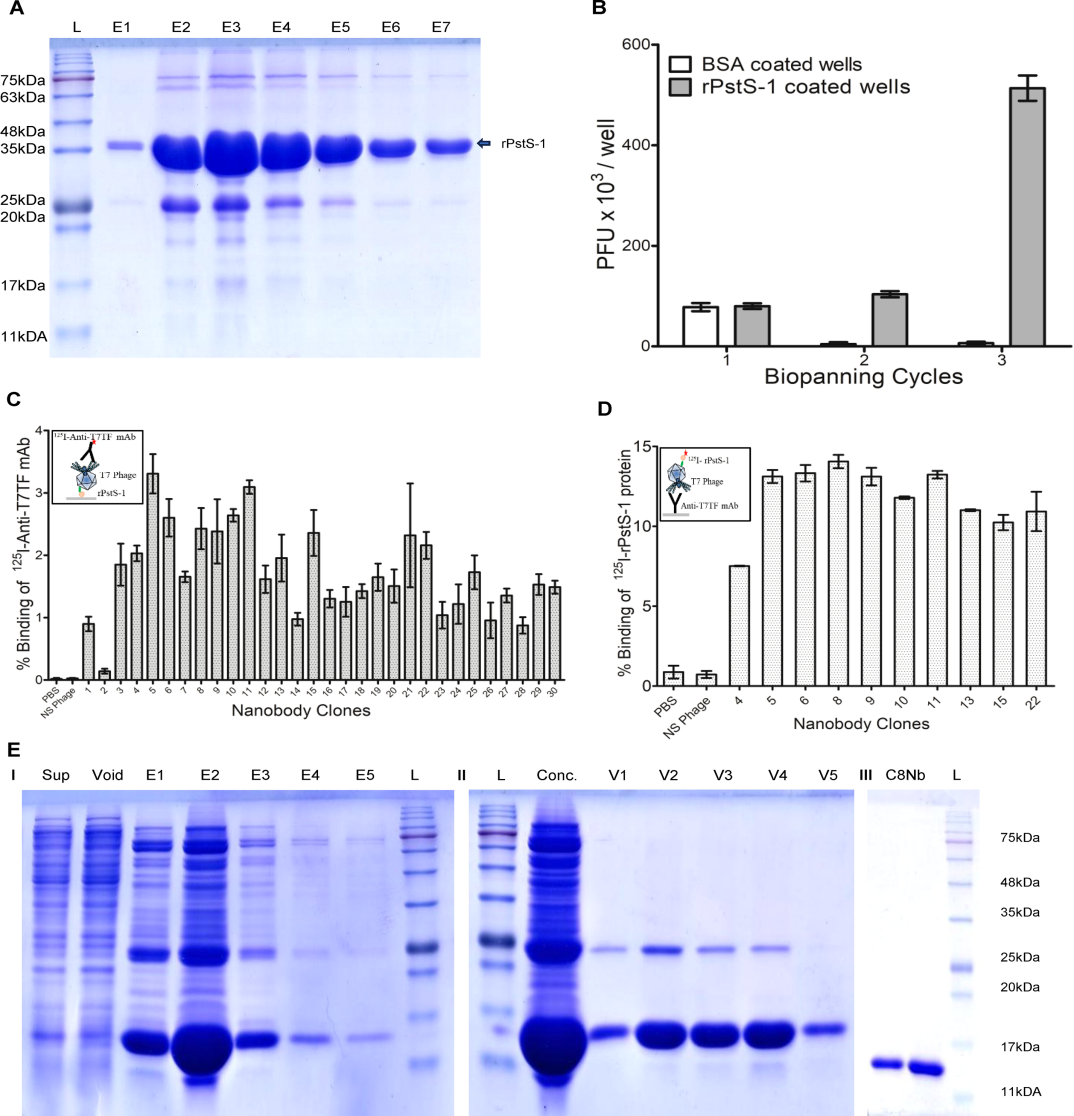
Isolation of nanobody specific to PstS-1 protein of Mtb. **(A)** 38kDa size rPstS-1 protein of Mtb was overexpressed in *E*. *coli* BL21(DE3) and soluble intact protein was purified using Ni-NTA agarose column chromatography. Lane 1- Protein marker; Lane 2–8 represents eluates from 1 to 7, respectively. **(B)** Three cycle bio-panning was performed to enrich PstS-1 protein specific nanobodies. Stringent washing produced more than 100-fold phage enrichment in rPstS-1 protein coated wells over BSA coated wells. **(C)** Nanobody screening was carried out using two assay formats. Immunoreactivity of randomly selected nanobody clones was evaluated by reacting monoclonal phage lysates with coated rPstS-1 protein and bound phages were measured by incubating ^125^I-anti-T7TF mAb. Out of 30 clones, 29 phages demonstrated strong positive binding. In both assays 1xPBS and non-specific (NS) phage were included as negative controls. **(D)** Reproducibility of immunoreactivity of nanobody clones which were selected in the first assay was confirmed by capturing phages in anti-T7TF mAb coated wells, followed by reacting wells with ^125^I labelled rPstS-1 protein. Image inserts in the graphs depict immunocomplex formation in screening assays. **(E)** C8Nb was overexpressed in *E*. *coli* BL21(DE3) and soluble protein was purified using (I) Ni-NTA agarose column chromatography and eluates (E1-E5) containing nanobody were pooled together, concentrated and subjected to (II) DEAE cellulose column chromatography. Void fractions (V1-V5) containing unbound C8Nb were pooled together. (III) 14.33kDa size C8Nb was further purified using size exclusion chromatography on Superdex G75 Increase 10/300 GL column.

Primary nanobody library was amplified in liquid culture and used for nanobody isolation. rPstS-1 protein was overexpressed and purified from *E. coli* BL21(DE3) in soluble form (**Figure 3A**). Three cycle bio-panning with stringent washing strategy removed weak binders and yielded >100 fold enrichment of nanobodies specific to rPstS-1 protein (**Figure 3B**). After enrichment, immunoreactivity of randomly selected 30 phage clones was assessed in two steps. In the first assay, 29 nanobody clones exhibited strong positive binding towards the coated rPstS-1 protein (**Figure 3C**). From these, ten nanobody clones that consistently demonstrated higher binding across three independent binding assays were selected for subsequent assay, which was performed to ensure that the binding obtained in first assay was specific to rPstS-1 protein. In the second assay, all phage lysates captured through phage tails, reproduced specific immunoreactivity with ^125^I-labelled rPstS-1 protein (**Figure 3D**). Sanger sequencing (data not shown) revealed that nanobody clone 8 and 11 were identical at the nucleotide level, while clone 5 had two transversions in FR1 region as compared to clone 8/11(C8Nb). Therefore, C8Nb was selected for the further studies and its variant carrying a C-terminal 6×His tag was overexpressed in *E. coli* BL21(DE3) in a soluble form. Subsequently, C8Nb was purified to single band using metal affinity chromatography, anion exchange chromatography and size exclusion chromatography sequentially (**Figures 3EI-III****).**

### High affinity C8Nb specifically recognizes PstS-1 protein

**Figure 4:**
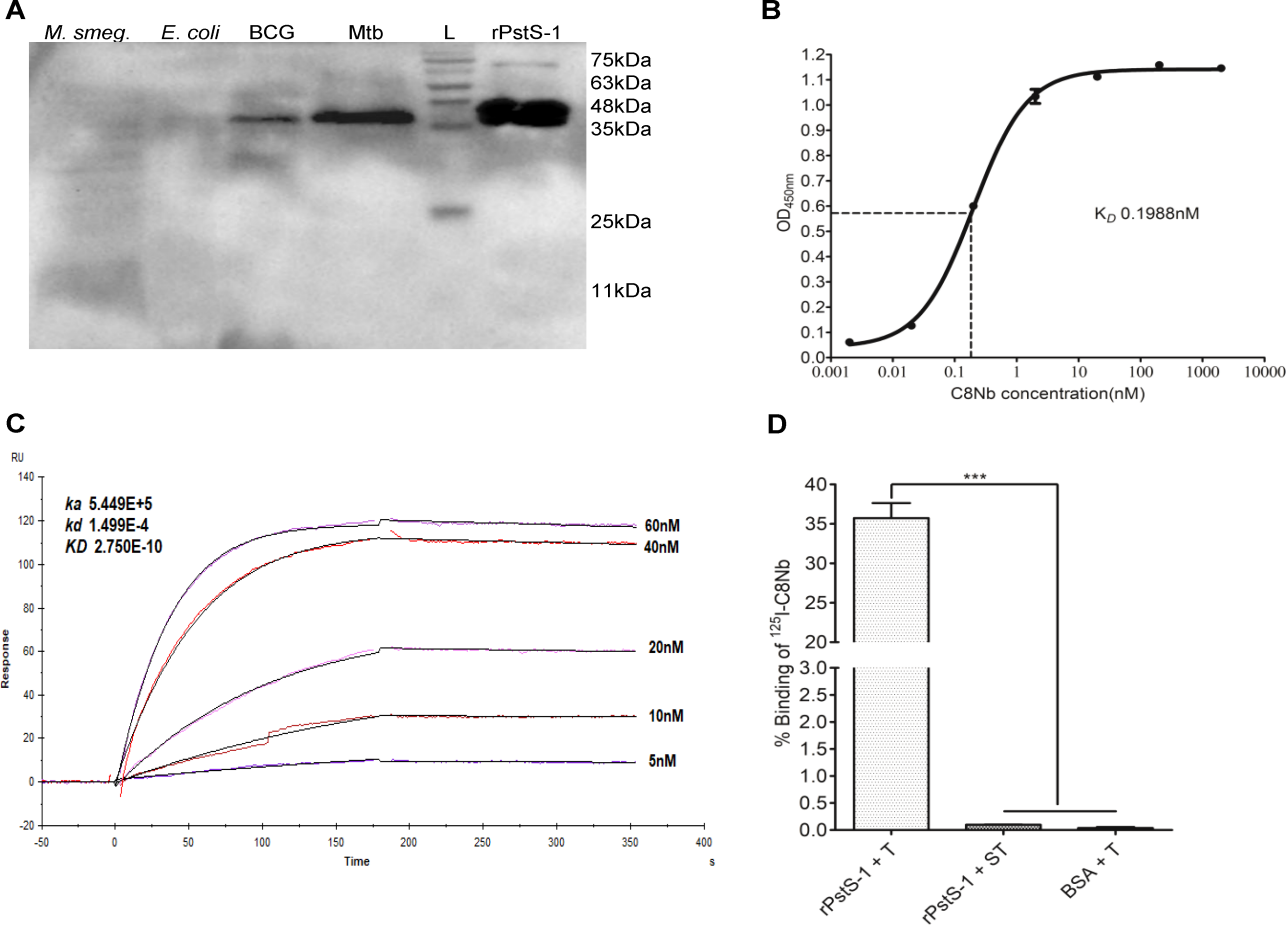
High affinity C8Nb specifically recognizes PstS-1 protein. **(A)** Bacterial protein mixtures or rPstS-1 protein were electrophoresed on SDS-PAGE and blotted on PVDF membrane, followed by sequential incubation with C8Nb and rabbit anti-camelid V_H_H-HRP conjugate. Band development around 38kDa size marker was observed only in the lanes loaded with either rPstS-1 protein or protein mixtures of Mtb and BCG. **(B)** Saturation binding ELISA demonstrated *K_D_* 0.198nM for C8Nb. **(C)** Binding of C8Nb to rPstS-1 was analyzed using a Biacore X100 SPR system. The rPstS-1 was immobilized on CM5 sensor chip and sensorgrams were obtained at increasing analyte (C8Nb) concentrations. Kinetic rate constants *k_a_* (5.44x10^5^ M^−1^ sec^−1^), *k_d_*(1.499x10^4^ sec^−1^) and the equilibrium dissociation constant *K_D_* (0.275nM) were determined using 1:1 binding model. **(D)** Immunoreactivity of ^125^I-C8Nb tracer (T) was confirmed against rPstS-1 protein coated in ELISA wells by radioimmunoassay. Spiked tracer (ST), prepared by mixing the tracer with excess unlabeled C8Nb, was included to assess specificity of binding. All values are mean ± SD. Significance was calculated by one-way ANOVA followed by Dunnett’s multiple comparisons *post hoc* test; ***P <0.001. Data are representative of more than three independent experiments.

Nanobody clone enrichment and screening were performed using the rPstS-1 protein, which contains few vector derived additional amino acid residues including a 6×His tag. As PstS-1 is a secretory protein, it may share conserved amino acid sequences involved in protein trafficking and interactions with cell wall components (44). Therefore, to ascertain that C8Nb specifically recognizes PstS-1 protein and no other proteins from Mtb lysate, we performed immunoblotting using C8Nb on PVDF membrane blotted with rPstS-1 and crude protein extracts from Mtb, BCG, *M. smegmatis* and *E. coli* (**Figure 4A**). Following sequential incubation with C8Nb, rabbit anti-camelid V_H_H antibody-HRP conjugate and CLS, an intense band around ~38kDa size appeared in the lane containing rPstS-1 protein. A single band of similar molecular weight was also observed in the lanes loaded with Mtb and BCG lysates, while no corresponding band appeared in the lanes containing *E. coli* or *M. smegmatis* proteins. A faint band near the 75kDa marker in the rPstS-1 lane, absent in other samples, likely represents a dimeric form of the rPstS-1 protein. Collectively, immunoblotting data suggests that the C8Nb specifically recognizes PstS-1 protein expressed by Mtb and BCG.

The affinity of C8Nb was evaluated by measuring *K_D_* using both saturation binding ELISA and SPR. In the ELISA, rPstS-1 protein coated wells were sequentially incubated with serially diluted C8Nb, rabbit anti-camelid V_H_H antibody-HRP conjugate and TMB substrate. Analysis of ELISA data revealed the *K_D_* value of 0.198nM (**Figure 4B**). SPR measurements using the Biacore X100 system (GE Healthcare) further characterized the binding kinetics between C8Nb and rPstS-1. C8Nb exhibited a rapid association with immobilized rPstS-1 with an association rate constant (*k_a_*) of 5.44×10^5^ M^-^¹s^-^¹ and a notably slow dissociation rate constant (*k_d_*) of 1.499×10^-4^ s^-^¹, as indicated by the nearly parallel curves in the dissociation phase of the sensorgrams. This exceptionally low dissociation rate is advantageous for molecular imaging applications. The *K_D_*, calculated as the ratio of *k_d_* to *k_a_*, was determined to be approximately 0.275nM (**Figure 4C**). To further validate the immunoreactivity of ^125^I-C8Nb against rPstS-1 protein, radioimmunoassay (RIA) was performed (**Figure 4D**). The ^125^I radionuclide was chosen for nanobody labelling due to the ease of labelling procedure and absence of particulate emissions. C8Nb was labelled with ^125^I radionuclide using Iodogen method and purified using size exclusion chromatography. When ^125^I-C8Nb (tracer) was incubated in wells coated with rPstS-1 protein, ~35.73 ± 3.29% of added tracer was in bound form, whereas in BSA coated control wells tracer binding was <0.05%. Moreover, when the rPstS-1 protein coated wells were reacted with spiked tracer, the binding of tracer was reduced to <0.1% of added tracer, which suggests that the immunoreactivity of C8Nb was retained after radioiodination.

### C8Nb recognizes PstS-1 protein present on the surface of drug sensitive as well as MDR strains of Mtb

**Figure 5:**
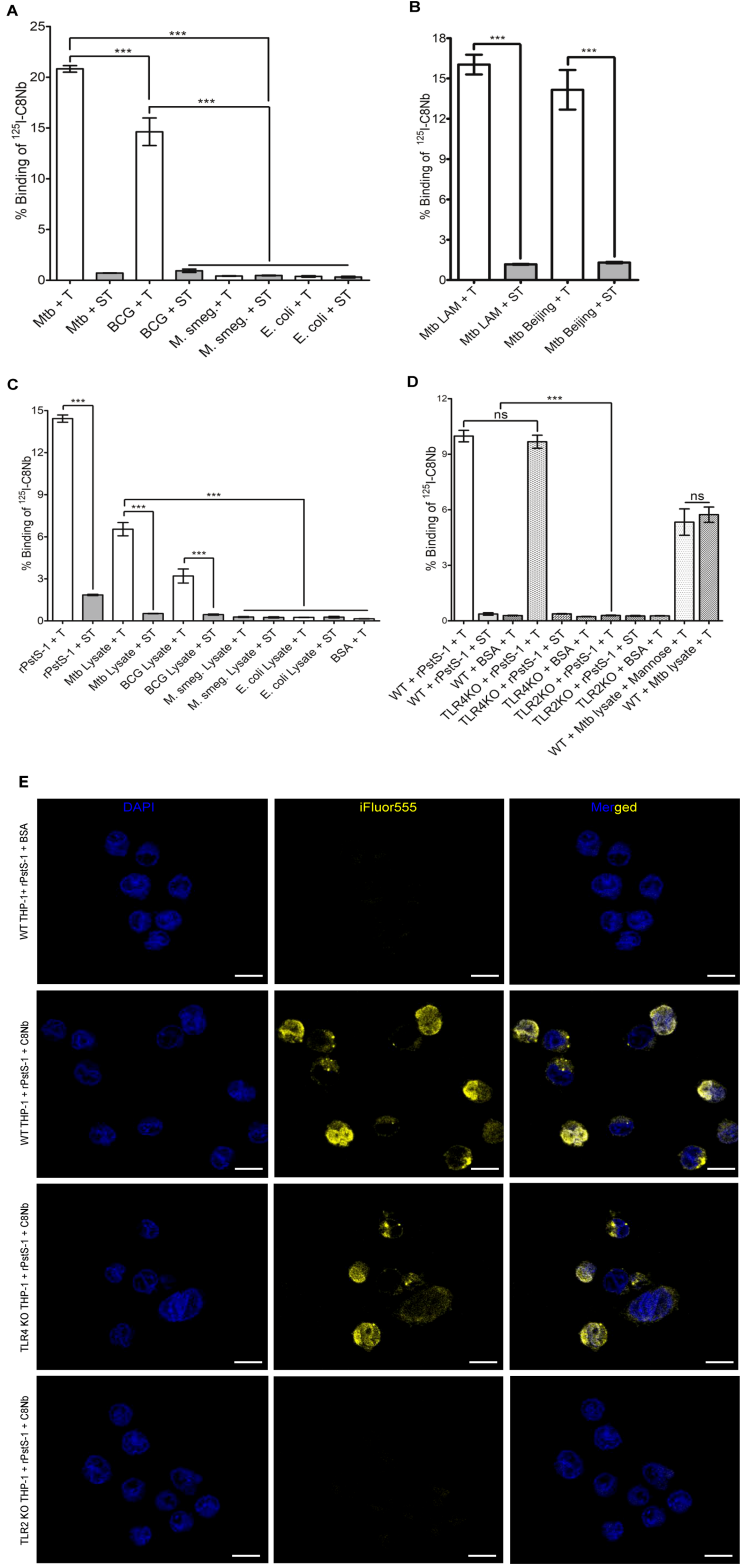
C8Nb recognizes PstS-1 protein adhered on the surface of either mycobacteria or macrophages. **(A)** When single cell suspensions of bacteria were probed with ^125^I-C8Nb, only Mtb and BCG pellets demonstrated tracer binding. **(B)** Immunoreactivity of ^125^I-C8Nb was confirmed with single cell suspensions of Mtb LAM and Mtb Beijing strains. **(C)** Protein mixtures prepared from bacterial lysates or rPstS-1 protein were adhered on formaldehyde fixed RAW 264.7 macrophages and then cells were incubated with ^125^I-C8Nb. Tracer showed binding with the cells which were treated with either rPstS-1 protein or protein mixtures prepared from Mtb or BCG lysates. **(D)** In similar binding studies on THP-1 macrophages adhered with rPstS-1 protein, WT and TLR4 KO cells demonstrated comparable tracer binding, while tracer binding with TLR2 KO cells was low and comparable with WT cells treated with BSA. In the case of MR, spiking Mtb protein mixture with mannose (1mg/ml) did not affect tracer binding. Data suggested that the epitope on PstS-1 protein bound to only TLR2 is accessible to C8Nb for binding, while MR and TLR4 are not playing role in binding. In all assays **(A-D)**, specific binding of ^125^I-C8Nb tracer (T) was assessed by including spiked tracer (ST). **(E)** In fluorescence microscopy, THP-1 cells were sequentially incubated with rPstS-1 protein, C8Nb and rabbit anti-camelid V_H_H cocktail-iFluor555. Analysis of acquired images suggested that TLR2 is involved in binding of C8Nb with PstS-1 protein treated THP-1 macrophages. Scale bars: 10μm. **(A-D)** all values are mean ± SD, and derived from more than three independent experiments. Significance was calculated by one-way ANOVA followed by Dunnett’s multiple comparisons or Bonferroni’s *post hoc* test; ***P <0.001.

In order to understand whether the epitope on PstS-1 protein adhered on the surface Mtb bacilli is accessible for C8Nb binding, we assessed the binding of ^125^I-C8Nb with mycobacteria. When the tracer was incubated with bacilli prepared from log phase cultures, the pellets of Mtb and BCG retained 20.82% ± 0.56 and 14.62% ± 2.36 of added tracer, respectively, while there was <0.5% tracer retention in the pellets of *M. smegmatis* and *E. coli*. Additionally, when the similar bacterial pellets were treated with spiked tracer, the binding of tracer was reduced to <1% (**Figure 5A**). Binding assay data indicated that the epitope of PstS-1 protein anchored on the surface of Mtb and BCG bacilli is accessible to C8Nb, and C8Nb did not recognize PstS-1 protein homologues expressed by *M. smegmatis* as well as *E. coli.* Using a similar assay, immunoreactivity of ^125^I-C8Nb with two MDR clinical isolates of Mtb belonging to distinct genetic lineages such as LAM (Latin American, Mtb LAM) and Beijing (Mtb Beijing) was also investigated and data suggested the binding of C8Nb with both the Mtb isolates (**Figure 5B**).

### C8Nb recognizes PstS-1 protein adhered on the surface of macrophages

Along with Mtb bacilli, immune cells surrounding the Mtb foci at infection site are key components of TB granulomas. These immune cells utilize various PRRs to recognize pathogen associated molecular patterns (PAMPs). The PstS-1 protein of Mtb is known to bind PRRs such as TLR2, TLR4 and MR, which are expressed on the surface of immune cells. To examine whether the PstS-1 protein bound to PRRs on RAW 264.7 macrophages could be recognized by C8Nb, an assay was performed using formaldehyde-fixed macrophages. Cells were treated with either rPstS-1 protein or bacterial protein mixtures followed by incubation with ^125^I-labeled C8Nb. Cells exposed with rPstS-1 protein, Mtb protein mixture and BCG protein mixture retained 14.42% ± 0.36, 6.54% ± 0.67 and 3.2% ± 0.71 of added tracer, respectively, indicating that C8Nb specifically recognizes the PstS-1 protein bound to macrophage surface receptors (**Figure 5C**). In contrast, the control wells, wherein cells were incubated with protein preparation from *E. coli, M. smegmatis* or BSA retained <0.5% of added tracer. Additionally, in the wells where cells were treated with similar protein mixtures prepared from bacterial lysates and spiked tracer, the binding of tracer was significantly reduced to <0.5%.

Collectively, these results demonstrate the specific interaction of C8Nb with the PstS-1 protein anchored on the macrophage surface.

### C8Nb recognizes PstS-1 protein bound to TLR2 but not to MR and TLR4

Additionally, we aimed to identify the PRR involved in tracer binding with macrophages treated with PstS-1 protein. We used WT as well as TLR2 and TLR4 KO THP-1 cell line derived macrophages (**Figure 5D**). When PMA activated THP-1 cells were fixed with formaldehyde and sequentially incubated with rPstS-1 protein and ^125^I-C8Nb, WT THP-1 cells expressing all the three PRR retained 9.98% ± 0.44 of added tracer, which was comparable with 9.68% ± 0.50 tracer binding with TLR4 KO cells. Similarly, WT THP-1 cells treated with mixture of Mtb proteins showed 5.34% ± 1.01 and 5.73% ± 0.58 of tracer binding with no significant effect from the presence or absence of mannose in the protein preparations. Furthermore, when similar antigen treated cells were incubated with tracer spiked with unlabeled C8Nb, the tracer binding was reduced to the levels similar to those seen with BSA treatment. These results suggested that neither MR nor TLR4 is involved in binding. However, in the case of TLR2 KO cells, tracer binding was reduced to the level observed in WT THP-1 cell treated with BSA, suggesting the involvement of TLR2 in the binding with PstS-1 protein-C8Nb complex. The role of TLR2 was further confirmed by confocal microscopy (**Figure 5E**). WT and TLR4 KO THP-1 cells treated with rPstS-1 protein and C8Nb showed binding of rabbit anti-camelid V_H_H cocktail-iFluor555 to a similar extent, whereas TLR2 KO THP-1 cells showed minimal binding of rabbit anti-camelid V_H_H cocktail-iFluor555, which was comparable with WT THP-1 cells not treated with C8Nb.

### ^125^I-C8Nb localizes into intramuscularly injected BCG cells in mouse

**Figure 6:**
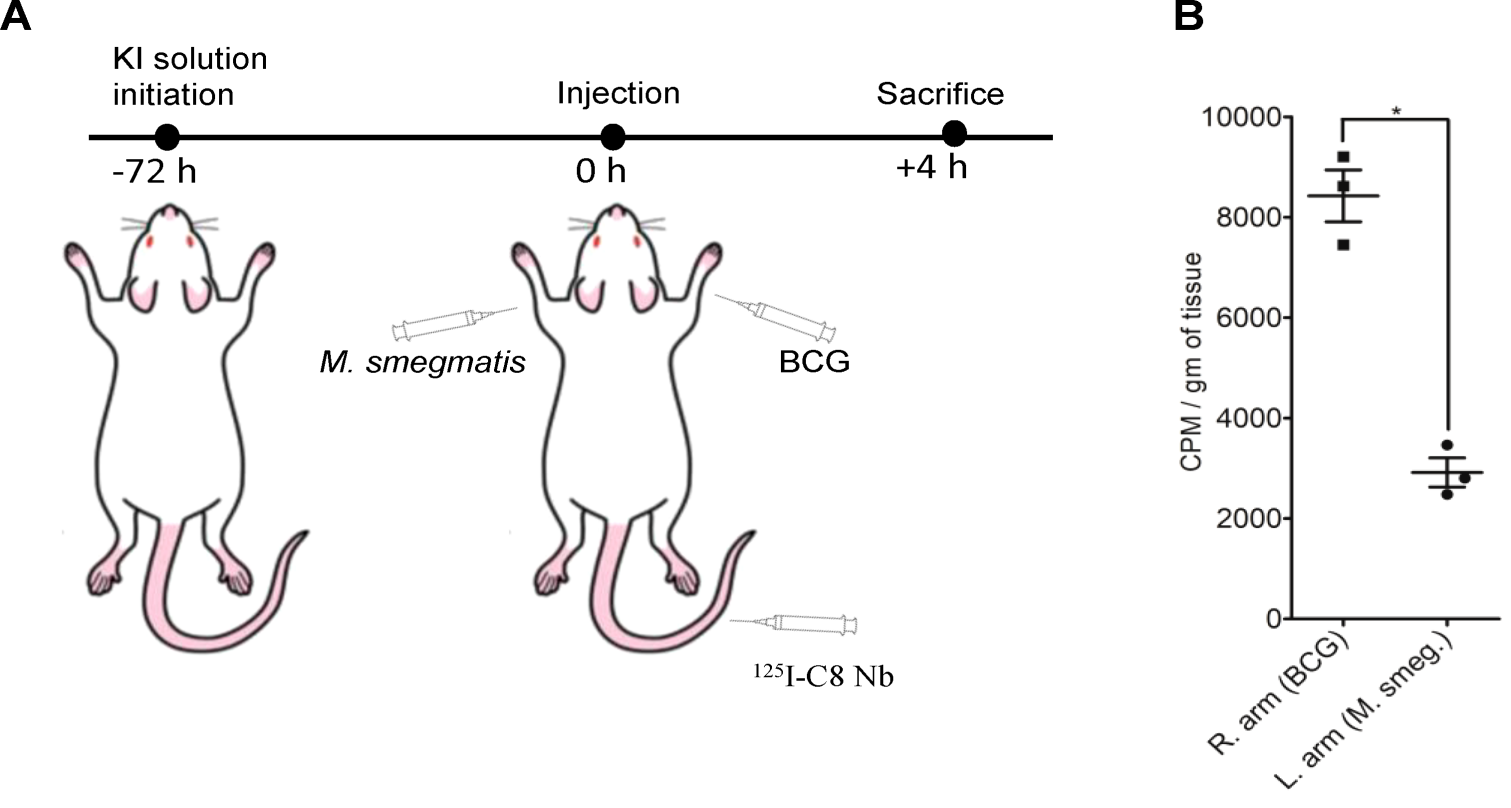
*In vivo* localization of ^125^I-C8Nb around intramuscularly injected mycobacterial cells. **(A)** To reduce uptake of free ^125^I in thyroid gland, BALB/c mice were fed with KI solution. For *in vivo* localization study, animals (n=3 animals) were injected with single cell suspensions of BCG (right forelimb) and *M. smegmatis* (left forelimb) through intramuscular route and 25.91µCi of ^125^I-C8Nb through catheterized tail vein. **(B)** Four hours post tracer administration, animals were sacrificed and limbs were dissected and accumulated activity was measured in gamma-photon counter. All mean ± SD. values are derived from more than three independent experiments. Significance was calculated by one tailed T-test; *P <0.05.

The binding properties of ^125^I-C8Nb to PstS-1 protein were further evaluated in BALB/c mice injected intramuscularly with single cell suspensions of BCG and *M. smegmatis* (**Figure 6A**). In the biodistribution study (**Figure 6B**), right forelimbs of mice injected with BCG retained 8427 ± 893 CPM/gm of tissue, which was significantly higher than the 2917 ± 503 CPM/gm of tissue retained in the left forelimbs inoculated with *M. smegmatis*. Radioactivity counts obtained in the hindlimbs, which were not inoculated with any bacterial cells, were consistently lower than any forelimbs containing bacterial pellets.

These results indicate that ^125^I-C8Nb selectively accumulates at sites of BCG infection, demonstrating its specific binding to PstS-1 *in vivo*.

## Discussion

Early diagnosis is one of the key components to reduce the global epidemic of TB to an endemic disease under ‘End TB strategy’ (2). Current TB diagnostic methods include multiple *in vitro* tests such as smear microscopy, microbial culture and various NAATs which often show inadequate sensitivity in samples with low bacillary load such as paucibacillary TB and EPTB (45, 46). Other tests including tuberculin skin test (TST) and IFN-gamma release assays (IGRA), which measure memory T cell response generated during the Mtb infection are less sensitive in immunocompromised individuals (47, 48). Conversely, humoral immune response against mycobacterial lipoarabinomannan is known to reduce the sensitivity of lateral flow immunochromatography in HIV negative TB patients (49).

In addition to *in vitro* diagnostics, non-invasive anatomical as well as molecular imaging have been pursued for TB diagnosis. The antigen-rich foci within TB granulomas represent suitable targets for antibody-based molecular imaging. Around the last decade of 20^th^ century, radiolabelled antibodies were evaluated for immunoscintigraphic detection of TB granulomas. Hazra et al. demonstrated immunoscintigraphic imaging of subcutaneously established Mtb infection in mouse using radiolabelled anti-PstS-1 protein monoclonal antibody (18). Similarly, Malpani et al. observed the earliest localization of ^131^I labelled anti-*M. bovis* BCG polyclonal antibodies in subcutaneous Mtb nodule after 3 days of intravenous injection, however, the best imaging contrast was developed only after 6 days (17). When, Lee et al. injected ^131^I labelled polyclonal anti-*M. bovis* BCG F(ab)_2_ fragments, best imaging contrast was developed within 24 h post tracer administration due to faster clearance of tracer from the circulation (19). While these initial studies highlighted the potential of radiolabelled antibody-based imaging of EPTB, the advent of highly sensitive PCR-based diagnostics reduced interest in this approach. However, renewed attention has emerged after realizing the urgent need of diagnostic tools for the management of paucibacillary TB and EPTB, leading to the development and evaluation of radiotracers like ^18^F-fluoro-deoxyglucose (FDG) (50), ^18^F-fluoro-deoxytrehalose (FTD) (51), and ^99^Tc-ethambutol (52) for TB diagnosis. Nevertheless, nonspecific nature of these tracers warrants further evaluation of Mtb antigen specific small antibody fragments which are known to quickly localize in target tissue and clear from the circulation which allows imaging within 2–4 h.

In this study, we report the isolation of a high-affinity nanobody (C8Nb) specific for the PstS-1 protein and evaluate its immunoreactivity with PstS-1 present on both Mtb bacilli and macrophages, the primary constituents of TB granulomas. With the goal of isolating and characterizing nanobodies specific to various antigenic targets having diagnostic potential in TB, a nanobody library against the full mixture of secreted Mtb proteins was constructed. The target antigen was chosen based on certain criteria such as; the secreted Mtb protein should remain adhered on the bacilli surface and if released, it should remain anchored on the surface receptors of host immune cells. PstS-1, a component of PST complex, composed of various protein subunits such as integral inner membrane proteins (PstA and PstC), cytoplasmic ATP hydrolyzing subunit (PstB) and periplasmic phosphate specific transport phosphate binding proteins (PstS); fulfils the criteria (27, 28). Mtb actively traffics PstS-1 protein (also known as 38-kDa antigen, PhoS, pab, Ag78, Ag5 or PhoS1), an immunodominant glyco-lipoprotein (29, 30), across the cell membrane where the protein remains bound to the cell wall (31) through its hydrophobic tail and participates in the phosphate transport (32). As an adhesion factor, PstS-1 protein interacts with MR on the macrophages to promote phagocytosis of Mtb (30), and its engagement with both TLR2 and TLR4 induces the ERK1/2 and p38 MAPK signaling in monocytes leading to TNF-α and IL-6 expression during mycobacterial infection (33, 34). Considering its anchoring properties and 38kDa size which can accommodate simultaneous binding of anchoring receptor and nanobody, we selected PstS-1 protein as a potential target for imaging of TB granulomas and isolated a high affinity C8Nb. Binding affinity of C8Nb was measured using ELISA and SPR, which revealed comparable values of *K_D_* as 0.198nM and 0.275nM, respectively. SPR results also provided insight into the real time binding kinetics (53). Immunoblot analysis confirmed that C8Nb specifically recognizes PstS-1 protein in the Mtb lysate. As the phosphorous is a very crucial macronutrient for growth, the PST complex appeared very early in the evolution and homologous systems are also reported in the other bacteria (54). On the same immunoblot, C8Nb also recognized PstS-1 protein homolog expressed by BCG, however, there was no nanobody binding and band development around the corresponding size in the lanes of *E. coli* and *M. smegmatis* (44, 55), suggesting the absence of epitope recognized by C8Nb.

C8Nb contains 6 tyrosine residues which are dispersed exclusively in the framework region-3. We labelled purified C8Nb with ^125^I using Iodogen method which attaches iodine atoms on the meta-position of benzene ring in tyrosine residues (56). When radiolabeled C8Nb was evaluated for its binding with bacterial single cell suspensions, pellets of Mtb and BCG retained 20.82% ± 0.56 and 14.62% ± 2.36 of added tracer, respectively. This binding data suggests that the epitope on PstS-1 protein which is present in mycobacterial cell wall is accessible for nanobody binding and hence imaging of Mtb bacilli or clumps in the granulomas is possible using C8Nb. Less tracer retention in the pellets of *E. coli* and *M. smegmatis* shows less or no binding of tracer which corroborated with the results of immunoblotting. Incubation of cell pellets with spiked tracer reduced the retention of tracer significantly, suggesting the specific tracer-antigen binding. Notably, C8Nb also detected PstS-1 protein of multi drug-resistant clinical isolates such as Mtb LAM and Mtb Beijing, supporting wide range diagnostic utility of C8Nb.

The granuloma forming immune cells express multiple PRRs including MR, TLR2 and TLR4, which are known to interact with PstS-1 protein (34). In addition to Mtb cell wall associated PstS-1, nanobody binding to antigen anchored on the surface of immune cells will help in granuloma imaging by enhancing signal intensity. When RAW macrophages, adhered with antigens were incubated with ^125^I-C8Nb, cells treated with rPstS-1 protein retained 14.42% ± 0.36 of added tracer, while cells treated with protein mixture isolated from Mtb or BCG retained 6.54% ± 0.67 and 3.2% ± 0.71 of added tracer, respectively, suggesting the accessibility of the epitope on PstS-1 protein to C8Nb. As compared to rPstS-1, cells treated with protein mixtures derived from Mtb and BCG showed less tracer binding, that can be attributed to the less availability of PstS-1 protein in protein mixture as well as competition of other ligands from the bacterial lysates for PRR. Tracer binding in cells treated with Mtb protein mixture suggests that the PstS-1 protein was able to bind macrophage surface receptors even in the presence of other proteins from protein mixture, which allows authors to speculate that the PstS-1 protein dissociated from Mtb bacilli can bind to immune cells in the TB granulomas and may serve as binding target for C8Nb. Cells treated with protein mixtures from *E. coli* and *M. smegmatis* exhibited low tracer binding which was comparable to BSA treated cells, which suggests that the C8Nb will be able to differentiate TB granulomas from other infections. Further, significant reduction in tracer binding on cells treated with similar antigens and incubated with spiked tracer confirmed the specificity of tracer binding.

Thereafter, we sought to determine which PRR binds PstS-1 protein in such a way that the antigen epitope is accessible for C8Nb binding. Based on previous studies, involvement of MR in the binding with PstS-1 protein-C8Nb complex was explored by incubating WT THP-1 cells with mannose mixed Mtb protein mixture and tracer (30). However, presence of mannose in the Mtb protein mixture did not produce any significant difference in the tracer binding, which suggests that either the epitope on MR bound PstS-1 protein is not accessible for C8Nb binding or MR is sufficiently competed out by other mannosylated molecules from Mtb lysate (57). Similarly, comparable binding of tracer with rPstS-1 protein treated WT and TLR4 KO THP-1 cells suggests that the epitope on PstS-1 protein bound to TLR4 is not accessible for binding. However, in the case of TLR2 KO THP-1 cells, the tracer binding was reduced to the level of BSA treated cells, which indicates that the PstS-1 protein bound to TLR2 can be recognized by C8Nb. Future studies could confirm these interactions using purified MR, TLR2, and TLR4 in ELISA. TB granulomas, composed of Mtb bacilli and surrounding host immune cells, carrying sufficiently accumulated target antigen provide an ideal *in vivo* model for nanobody localization. Additionally, in the TB granulomas, Mtb bacilli released from necrotic macrophage may carry more copies of PstS-1 protein produced to survive in the starving phagosomal niche (31, 58). *In vitro* binding assays performed in this study, have shown stronger immunoreactivity of C8Nb with Mtb origin PstS-1 protein, either associated with cell wall or present in protein mixture, as compared to the protein homolog produced by BCG. This difference in C8Nb binding may be attributed to the differences in PstS-1 protein expression levels or binding affinity. Due to BSL-III restrictions we used BALB/c mice with intramuscularly injected BCG for *in vivo* study. The biodistribution data demonstrated significantly higher tracer localization in forearms inoculated with BCG cells than the other arms carrying *M. smegmatis.* Surprisingly, *M. smegmatis* inoculated limbs showed slightly higher tracer retention than uninjected hind limbs. Similar phenomenon was earlier reported by Lee et al, wherein radiolabeled BCG-specific F(ab’)2 showed initial hot uptake in syphilitic lesion of *Treponema pallidum* in rabbit followed by a rapid washout of the tracer (19). Increase in tracer localization in this case may be attributed to the enhanced influx of protein within the expanded interstitial fluid space and a reduced rate of clearance in inflammation (19). Studying the rate of influx into and efflux from the site of inflammation at various time points will be crucial as it will decide ideal time window for imaging.

Though, previous studies reported tracer localization in Mtb nodule even in the presence of immune response against injected antigens (17–19), in this study, presence of unlabeled C8Nb in the spiked tracer reduced the tracer binding significantly. Therefore, it will be crucial to study the effect of humoral immune response on *in vivo* tracer accumulation in TB granulomas, as the immunodominant PstS-1 protein is known to induce strong antibody response in TB patients (59). In earlier studies, Malpani et al. and Lee et al. reported tracer localization in nodules of sonicated Mtb lysate, which suggests that antibody based imaging may not be able to discriminate between live and dead Mtb bacilli. (17, 19). Therefore, to evaluate nanobody based molecular imaging for treatment response monitoring and predicting disease relapse, it will be crucial to study the impact of drug therapy initiation on tracer localization within TB granulomas.

## Data Availability

The raw data supporting the conclusions of this article will be made available by the authors, without undue reservation.

## Supplementary material

Supplementary Figure 1 Nanobody library construction using T7 phage display system. **(A)** Mature mRNAs depicting genetic elements of camel IgG immunoglobulin subclasses. **(B)** Partial genetic map of T7Select1-1b vector DNA along with EcoRI and HindIII sites within gene10. **(C)** Sticky overhangs of EcoRI and HindIII digested T7Select1-1b vector arms and V_H_H gene inserts. Amino acid sequence represents the modified gene10 ORF following cloning.
