# Supplementary figures and images for: Isolation of a nanobody specific to the PstS-1 protein and evaluation of its immunoreactivity with structural components of Mycobacterium tuberculosis granuloma
Source: Front Immunol. 2025 Dec 16;16:1684904. doi: 10.3389/fimmu.2025.1684904 (PMC12748192; doi:10.3389/fimmu.2025.1684904)

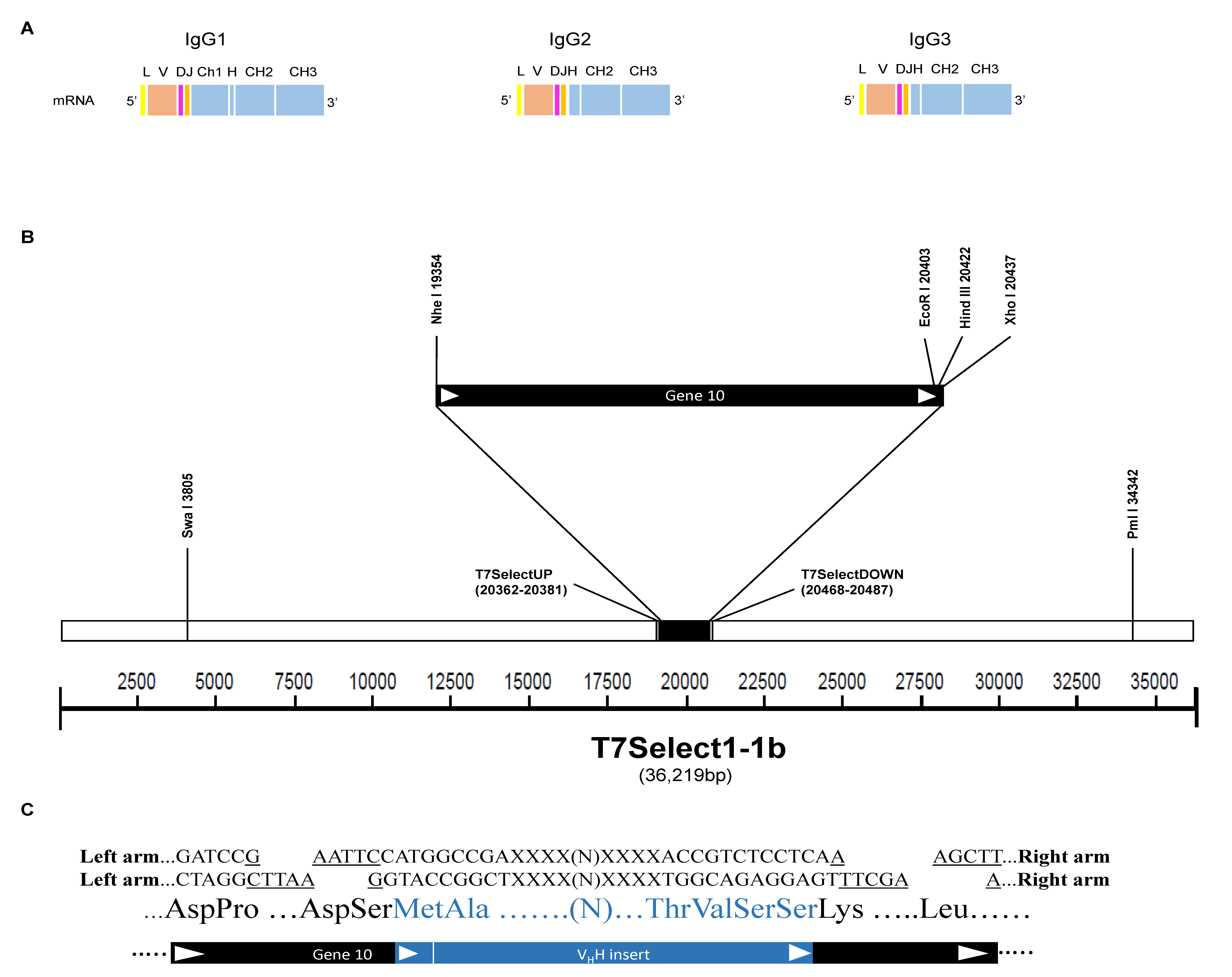

Supplement: Supplementary Figure 1 — Nanobody library construction using T7 phage display system. (A) Mature mRNAs depicting genetic elements of camel IgG immunoglobulin subclasses. (B) Partial genetic map of T7Select1-1b vector DNA along with EcoRI and HindIII sites within gene10. (C) Sticky overhangs of EcoRI and HindIII digested T7Select1-1b vector arms and VHH gene inserts. Amino acid sequence represents the modified gene10 ORF following cloning. [file Image1.tif]
