# Supplementary material for: Isolation of a nanobody specific to the PstS-1 protein and evaluation of its immunoreactivity with structural components of Mycobacterium tuberculosis granuloma
Source: Front Immunol. 2025 Dec 16;16:1684904. doi: 10.3389/fimmu.2025.1684904 (PMC12748192; doi:10.3389/fimmu.2025.1684904)
Supplement: Supplementary file 2 [file DataSheet1.pdf]

Supplementary Material

Table S1. List of DNA sequences.

Name	Sequence	Ref.
CH2FORTA4	5′ - CGCCATCAAGGTACCAGTTGA - 3′	(21)
VHBACKA6	5′ - GATGTGCAGCTGCAGGCGTCTGG(A\G)GGAGG - 3′	
FORECORI	5′ - GCCACTGAATTCCATGGCCGA(G/T)GT(G/C)CA GCT - 3′	
BACKHINDIII	5′ - GGACTAAAGCTTTGAGGAGACGGTGACCTG - 3′	
T7SelectUp	5′ - GGAGCTGTCGTATTCCAGTC - 3′	
T7SelectDown	5′ - AACCCCTCAAGACCCGTTTA - 3′	
C8Nb	atggccgatgtccagctgcaggcgtctggaggaggctcggtgcaggctggagggtctctgagactctcctgttcagcctctggagtcaccgccacagacgcctgcgtgggctggttccgccagtctccagggaaggagcgtgagacagtcgcgcacataaatactgcggatggtaagaactattacgccaattctgtgaagggccgattcaccatctcccaggacaacgccaagaacacggtgtatctgcaaatggacagcctgaaacttgaggattcggccatctattactgtgcggccagagaaggcagggtgttcggcttgggtgcgcccaacttatttaactactggggccaggggatccaggtcaccgtctcctca	DDBJ Accession No. **LC899821**
